# Rotavirus spike protein ΔVP8* as a novel carrier protein for conjugate vaccine platform with demonstrated antigenic potential for use as bivalent vaccine

**DOI:** 10.1038/s41598-021-01549-z

**Published:** 2021-11-11

**Authors:** Wook-Jin Park, Yeon-Kyung Yoon, Ji-Sun Park, Ruchirkumar Pansuriya, Yeong-Jae Seok, Ravi Ganapathy

**Affiliations:** 1 School of Biological Sciences and Institute of Microbiology, Seoul National University, Seoul, 08826 Republic of Korea; 2 International Vaccine Institute, Seoul, 08826 Republic of Korea

**Keywords:** Infectious diseases, Vaccines

## Abstract

Conjugate vaccine platform is a promising strategy to overcome the poor immunogenicity of bacterial polysaccharide antigens in infants and children. A carrier protein in conjugate vaccines works not only as an immune stimulator to polysaccharide, but also as an immunogen; with the latter generally not considered as a measured outcome in real world. Here, we probed the potential of a conjugate vaccine platform to induce enhanced immunogenicity of a truncated rotavirus spike protein ΔVP8*. ΔVP8* was covalently conjugated to Vi capsular polysaccharide (Vi) of *Salmonella* Typhi to develop a bivalent vaccine, termed Vi-ΔVP8*. Our results demonstrated that the Vi-ΔVP8* vaccine can induce specific immune responses against both antigens in immunized mice. The conjugate vaccine elicits high antibody titers and functional antibodies against *S*. Typhi and Rotavirus (RV) when compared to immunization with a single antigen. Together, these results indicate that Vi-ΔVP8* is a potent and immunogenic vaccine candidate, thus strengthening the potential of conjugate vaccine platform with enhanced immune responses to carrier protein, including ΔVP8*.

## Introduction

Rotaviruses are a primary cause of acute gastroenteritis in infants and children under five years of age, resulting in 24 million outpatient visits, 2.3 million hospitalizations, and 200,000 deaths annually^1–3^. Among several licensed rotavirus vaccines, four live attenuated oral vaccines (RotaTeq, Rotarix, ROTAVAC and ROTASIIL) are currently included in national immunization schedules in >100 countries worldwide^4^. While these vaccines  have been highly effective with 80–90% efficacy shown in developed countries, their efficacies are impaired in low- and middle-income countries where rotavirus vaccines are mostly needed^5–10^. Malnutrition, micronutrient deficiencies, and enteric co-infection may contribute to the diminished immunogenicity and effectiveness of those attenuated vaccines^11^. In addition, changes in rotavirus epidemiology may be another possible reason for the reduced vaccine outcomes^12^. Furthermore, the live attenuated vaccines may be associated with a low-level risk of intussusception^13^.

Non-replicating rotavirus vaccines for parenteral use have shown to be less prone to suboptimal effectiveness or unanticipated adverse events^14^. These vaccines potentially lead to enhanced efficacy in resource-deprived countries because they are not directly affected by microbiome composition or gut enteropathy^15^. Multiple studies have reported that a truncated rotavirus VP8* (ΔVP8*) subunit protein, from the proteolytic cleavage of the outer capsid spike protein VP4, is the target antigen due to its ability to induce highly potent neutralizing antibodies which confer strong protection against RV^16,17^. However, many defined neutralizing antigens, including ΔVP8*, possess low immunogenicity due to their small sizes with low valences. To this end, a large, multivalent vaccine platform has been developed that could make it possible for small viral or bacterial antigens to be more immunogenic^18–21^. For example, ΔVP8* has been used in several vaccine platforms including recombinant fusion proteins (ΔVP8* fused to a universal tetanus toxin CD4^+^ T cell epitope P2) and nanoparticles (ΔVP8*inserted in the surface loops of the Norovirus P or S particle)^22–24^.

In this study, we used a conjugate vaccine platform, wherein a carbohydrate moiety is covalently linked to a carrier protein, to increase the immunogenicity of the ΔVP8*. Although polysaccharide-protein conjugate vaccines were developed to enhance the immunogenicity of natural carbohydrate antigens, which are considered T cell-independent, a carrier protein may also induce an immune response against itself^25,26^. Therefore, the conjugate vaccine platform may induce protective immunity against the pathogen not only from which the polysaccharide is derived, but also from which the carrier protein is derived^25^. The virulence capsular polysaccharide (Vi) of *Salmonella* serovar Typhi (*S*. Typhi) is the target of a protective humoral immune response. Conjugation of Vi to a carrier protein can effectively convert a T cell-independent immune response to a T cell-dependent immune response, characterized by IgM-to-IgG switching, a booster response, and sustained T cell memory to Vi^27,28^. Vi-recombinant non-toxic form of *Pseudomonas aeruginosa* exotoxin A (rEPA) and Vi-Tetanus Toxoid (TT) conjugate vaccines have been licensed and marketed in China and India^29,30^, while Vi-Diphtheria Toxoid (DT) and Vi-a nontoxic diphtheria cross-reacting material (CRM_197_) are undergoing clinical trials for use in infants and children^31,32^.

Currently licensed conjugate vaccines use a few carrier proteins [(*i.e.*). TT, DT, CRM_197_, or Outer Membrane Protein Complex (OMPC)]^33^. The limited number of carrier proteins implies repeated exposure to the same carrier, causing various immune interference such as carrier-induced epitope suppression (CIES), carrier-specific enhancement of T cell help, and bystander interference^33,34^. In light of this, there is a need for alternative carrier proteins to overcome the impediments related to immune interference. Among several candidates, rEPA and a rationally designed recombinant protein containing strings of universal CD4^+^ T-cell epitopes proved to be good candidates as carrier proteins^35^.

RV is classified into 35 G and 50 P types determined by rotavirus surface proteins VP7 (glycoprotein) and VP4 (protease-sensitive) protein, respectively^36^. P[8] (Wa strain) and P[4] (DS-1 strain) are the most common P types causing more than 91% of infections in both developed and developing countries^37^. In Africa, however, P[6] (1076 strain) accounts for almost one-third of all P types^38^. Thus, a multivalent vaccine formulation consisting of ΔVP8* from P[4], P[6] and P[8] will provide broad efficacy against the dominant circulating P type rotavirus worldwide^37^. We developed purification steps for ΔVP8* antigens of P[4], P[6], and P[8] strains using two steps of ion-exchange chromatography. Different conjugation strategies were tested to improve vaccine design. The Vi-ΔVP8* conjugates were characterized physicochemically, and then mixed as a multivalent vaccine. Antigen-specific antibodies in serum bactericidal activity against *S*. Typhi and virus-neutralizing antibody response against RV determined the immunologic properties of Vi-ΔVP8* conjugates in mice. The results showed that the Vi-ΔVP8* conjugates induced significantly high immune response toward the ΔVP8* antigens and Vi polysaccharides, thus making it a promising multivalent vaccine candidate against multiple rotavirus P types and *S*. Typhi. Overall, our data shed light on an additional potential of a conjugate vaccine platform for enhanced immunogenicity of ΔVP8*.

## Results

### Expression, purification and specificity of ΔVP8*

**Figure 1:**
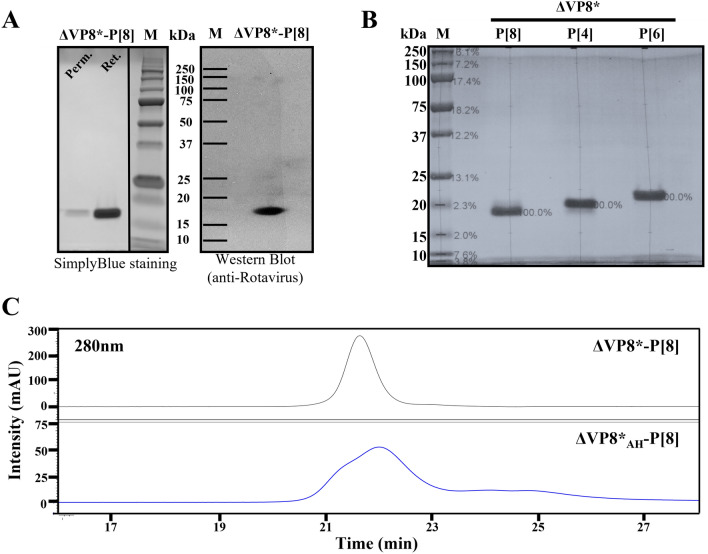
Production and physicochemical characteristics of the recombinant ΔVP8* proteins. (**A**) SimplyBlue stained SDS-PAGE (Ret.: Retentate from 5 kDa TFF, Perm.: Permeate from 5 kDa TFF, and M: Marker) and Western blot analysis of ΔVP8*-P[8] probed with anti-Rotavirus antibody (AB1129). The grouping of gels cropped from different parts of the same gels. The full-length blots/gels are presented in Supplementary information. (**B**) SimplyBlue stained SDS-PAGE of the three purified ΔVP8*. (C) SEC-HPLC analysis of purified ΔVP8*-P[8] before and after ADH derivatization. OHpak SB-804 HQ column, 0.1 M NaCl, 0.1 M NaH_2_PO_4_, 5% ACN, pH 7.2; 0.3 mL/min. flow rate.

The recombinant ΔVP8* protein with P[4], P[6] or P[8] specificity was expressed in the (*E. coli*) system. The IPTG-induced bacteria were then collected by centrifugation and lysed to release soluble proteins. The ΔVP8* proteins were purified by two-step ion exchange chromatography (Supplementary Fig. S1). The yields of three recombinant proteins reached ~50 mg/L of culture. After purification, all the ΔVP8* proteins showed a single band of 18–20 kDa on SDS-PAGE and was eluted as a single peak in SEC-HPLC (Fig. 1). The residual nucleic acid and endotoxin levels were 0.4 ng/mL and 1.84 EU/mL, respectively, which are within the acceptable levels for recombinant subunit vaccines.

Aspartic or glutamic acid residues of the purified ΔVP8* were modified with ADH in the presence of EDAC to achieve an efficient coupling of ΔVP8* to Vi. Derivatization of ΔVP8* (ΔVP8*_AH_) resulted in four to six ADH linkers per ΔVP8* (Table 1). The levels of modification were determined by TNBS assay. ΔVP8*_AH_ showed a broad peak at similar retention time on SEC-HPLC as the ΔVP8*, indicating no cross-linking of the VP8* had occurred (Fig. 1C).

**Table 1:** Characterization of Vi-ΔVP8* conjugates.

Conjugates	ADH linkers per protein	Recovery (%)		Average size (kDa)	Vaccine Formulation	
		**Vi**	ΔVP8*		**Vi (μg)**	ΔVP8* (μg)
Vi-ΔVP8*-P[4]	4	35	10	2665	5	1.0
Vi-ΔVP8*-P[6]	6	33	11	2583	5	1.1
Vi-ΔVP8*-P[8]	5	44	15	2773	5	1.5
Multivalent formulation	–	–	–	–	15	3.6

^1^Recovery of each conjugate obtained was calculated on the basis of Vi concentration and protein concentration.

^2^Vi concentration was based on Hestrin assay.

^3^ΔVP8* protein concentration was based on Bradford assay.

^4^Multivalent formulation is the mixture of Vi-ΔVP8*-P[4], P[6], and P[8].

### Chemical conjugation of ΔVP8*_AH_ to Vi

**Figure 2:**
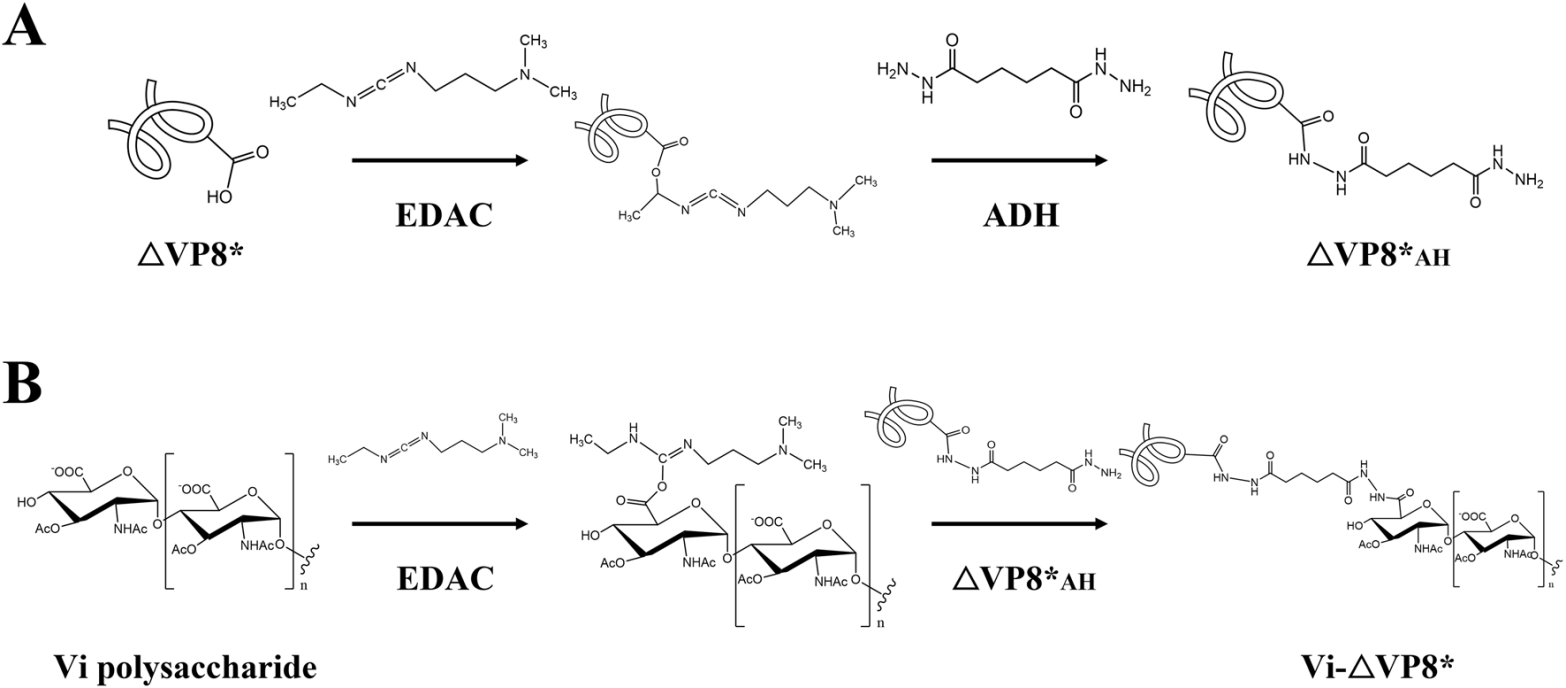
Schematic representation of the preparation of Vi-ΔVP8*. (**A**) ΔVP8* was derivatized with EDAC/ADH. (**B**) Vi polysaccharide was conjugated with ΔVP8*_AH_.

**Figure 3:**
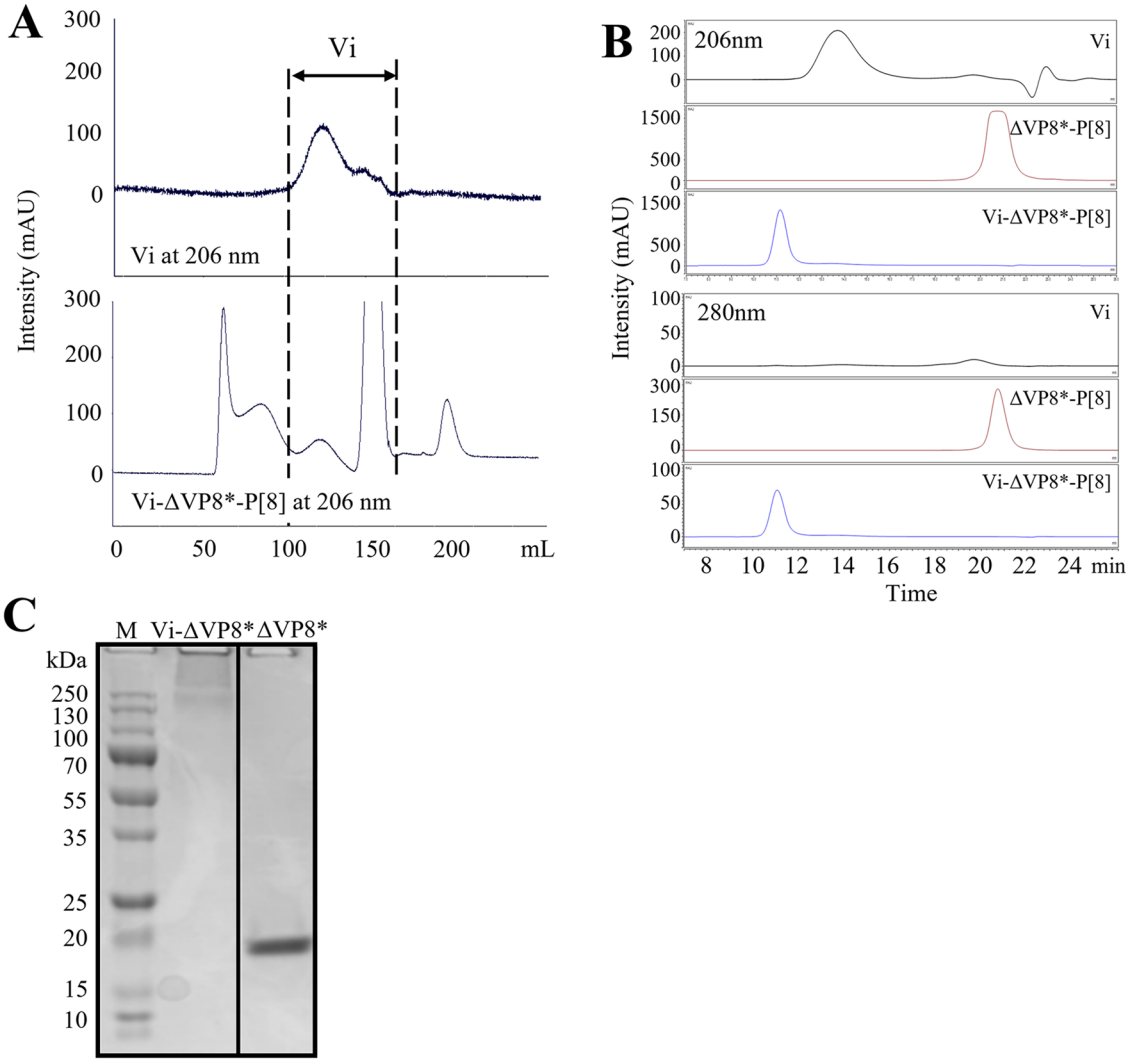
Physicochemical characteristics of the glycoconjugates. (**A**) Sephacryl S-1000 profile of Vi and Vi-ΔVP8*-P[8]. Vi-ΔVP8*-P[8] was purified on Sephacryl S-1000 (1.6 cm × 90 cm) eluting with 10 mM NaH_2_PO_4_, 5 mM NaCl, pH 7.0 at a flow rate of 0.5 mL/min. (**B**) SEC-HPLC profiles of Vi-ΔVP8*-P[8]. TSKgel G5000 PW_XL_ column, 0.1 M NaCl, 0.1 M NaH_2_PO_4_, 5% ACN, pH 7.2; 0.5 mL/min. flow rate. Vi polysaccharide, ΔVP8*-P[8], and Vi-ΔVP8*-P[8] (**C**) SDS-PAGE profile of Vi-ΔVP8*-P[8] (Lane 1: Marker, Lane 2: Vi-ΔVP8*-P[8] and Lane 3: ΔVP8*-P[8]). 12% gel, SimplyBlue staining. The grouping of gels cropped from different parts of the same gels. The full-length gels are presented in Supplementary information.

As illustrated in Fig. 2, glycoconjugates were synthesized with 8 mg of Vi (2 mg/mL) at a ratio Vi/ΔVP8*_AH_ of 1:1 (w/w) and EDAC (50 mg/mL) in MES buffer at pH 5.6. The chromatogram profile showed that Vi-ΔVP8* eluted earlier than either free Vi or free ΔVP8*, confirming the larger size of the conjugate with respect to free components (Fig. 3A and Supplementary Fig. S2A). The large size of the conjugates resulted in decreased retention time in SEC-HPLC (Fig. 3B and Supplementary Fig. S2B), and a typical smear appeared at the top of 12% SDS-PAGE (Fig. 3C). The recoveries of Vi and ΔVP8* were ~ 40% and ~ 10%, respectively (Table 1). As illustrated in Supplementary Fig. S3A, an attempt to directly link ΔVP8* to Vi was tried through the reaction between the primary amine in ΔVP8* and carboxylic groups along the Vi polysaccharide, previously activated with EDAC. However, the direct conjugation method was not successful. The limited number or low reactivity of primary amine in ΔVP8* could be one reason, and steric hindrance could be another. Alternatively, N-β-maleimidopropionic acid hydrazide (BMPH) was tried to facilitate coupling ΔVP8* to Vi. For this, ΔVP8* was firstly thiolated by N-succinimidyl S-acetylthioacetate (SATA) to introduce thiol groups, which were reacted with the maleimide groups in heterobifunctional linker, BMPH; the hydrazide groups left on the other side in the BMPH were further coupled with the carbonyls on Vi polysaccharide (Supplementary Fig. S3). However, the recovery of ΔVP8* in Vi-BMPH-ΔVP8* conjugates was lower (5%) than that obtained in Vi-ADH-ΔVP8* conjugates (10%) (Supplementary Table S1).

### Immunogenicity of Vi-ΔVP8* conjugates in mice

**Figure 4:**
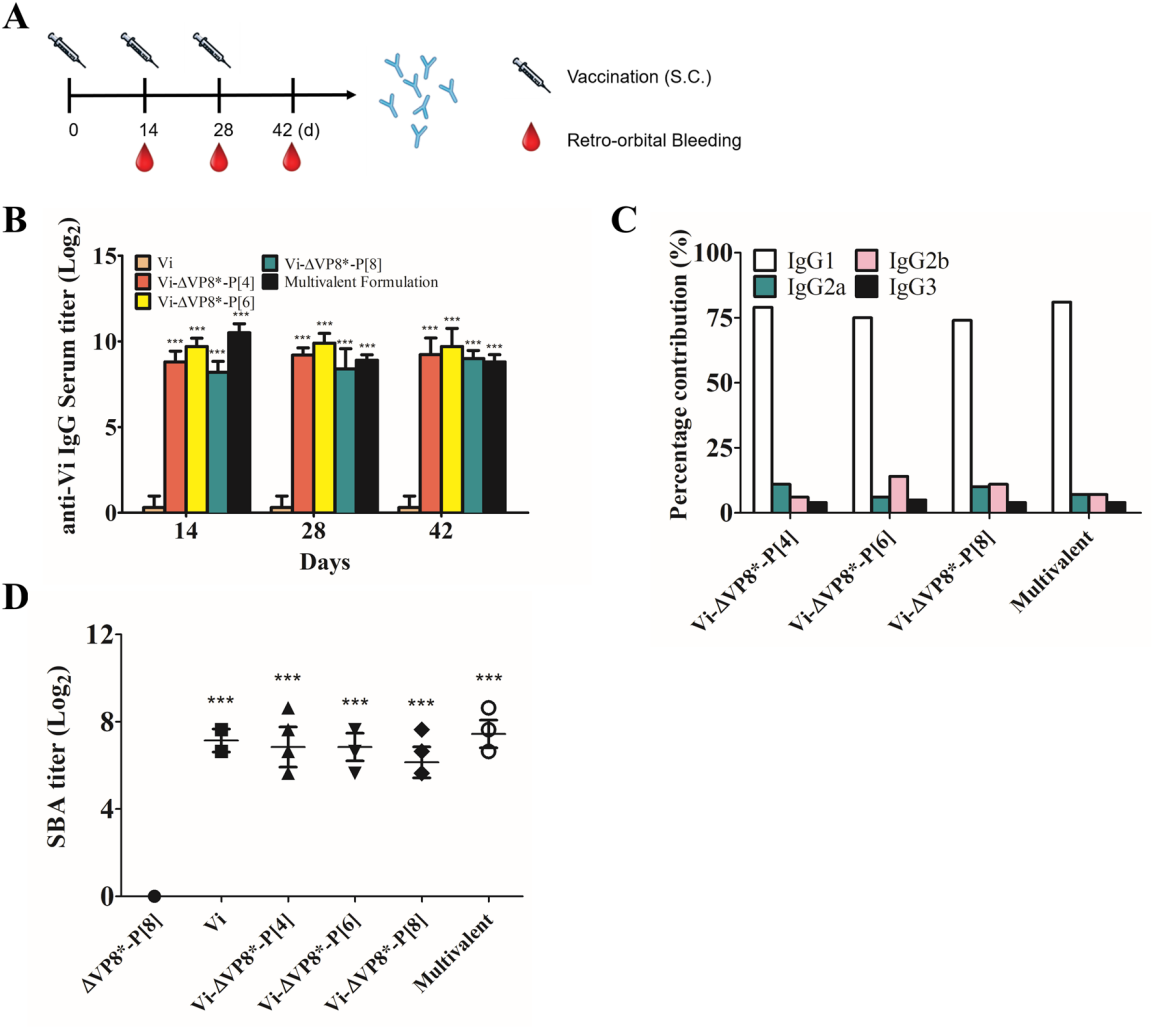
Immunogenicity study with Vi-ΔVP8* for anti-Vi. (**A**) A schematic diagram for subcutaneous (S.C.) vaccination and bleeding. (**B**) Anti-Vi antibody titer of conjugates. (**C**) Percentage contribution of IgG subclasses to total IgG against Vi. (**D**) Serum bactericidal activity (SBA) titers observed for sera from mice immunized with Vi-ΔVP8* conjugates. BALB/c mice were immunized SC with 5 μg of Vi-ΔVP8*-P[4], P[6] or P[8], or 15 μg of multivalent formulation based on Vi polysaccharide 3 times at 2-week intervals as described in Methods. Data were analyzed and illustrated using GraphPad Prism 6.0 software (GraphPad, https://graphpad.com); data are mean±s.d.; two sample *t*-test; *P<0.05, **P<0.01, *** *P*<0.001.

In immunogenicity study, Vi-ΔVP8* conjugates were administrated to mice without an adjuvant to focus exclusively on the intrinsic value of ΔVP8* as a carrier. As illustrated in Fig. 4A, mice were subcutaneously injected with each conjugate (5 μg Vi/dose) or multivalent formulation (15 μg Vi/dose) on days 0, 14 and 28. Four different groups of mice were injected with 5 μg of Vi, 1.0 μg of ΔVP8*-P[4], 1.1 μg of ΔVP8*-P[6], 1.5 μg of ΔVP8*-P[8]. Controls were injected with PBS. Serum anti-Vi IgG titers and functional antibody levels were measured by ELISA and complement-mediated SBA, respectively. Immunization with the unconjugated Vi induced very low levels of Vi-specific IgG antibodies, and it was consistent with our previous results that antisera raised against Vi alone in PBS elicited low titers in ELISA^39,40^. In contrast, the glycoconjugates induced highly elevated titers of IgG antibodies to Vi after a single immunization (Fig. 4B). The high titers observed on Day 14 persisted through Day 42. IgG1 was consistently the dominant IgG subclass against Vi in the immunized mice (Fig. 4C). Diluted serum samples were incubated with (*S.*). Typhi to determine bactericidal activity of sera from vaccinated mice. The serum from mice immunzied with Vi alone or Vi-VP8* conjugates showed a significant ability to prevent the growth of (*S.*)﻿. Typhi more than 50% until reaching 1:200 dilution, while sera from VP8* group showed no SBA titer (Fig. 4D and Supplementary Fig. S4). These data indicated that Vi-ΔVP8* conjugates elicited high level of anti-Vi IgG and complement-mediated SBA responses against (*S.*). Typhi isolate C6524.

**Figure 5:**
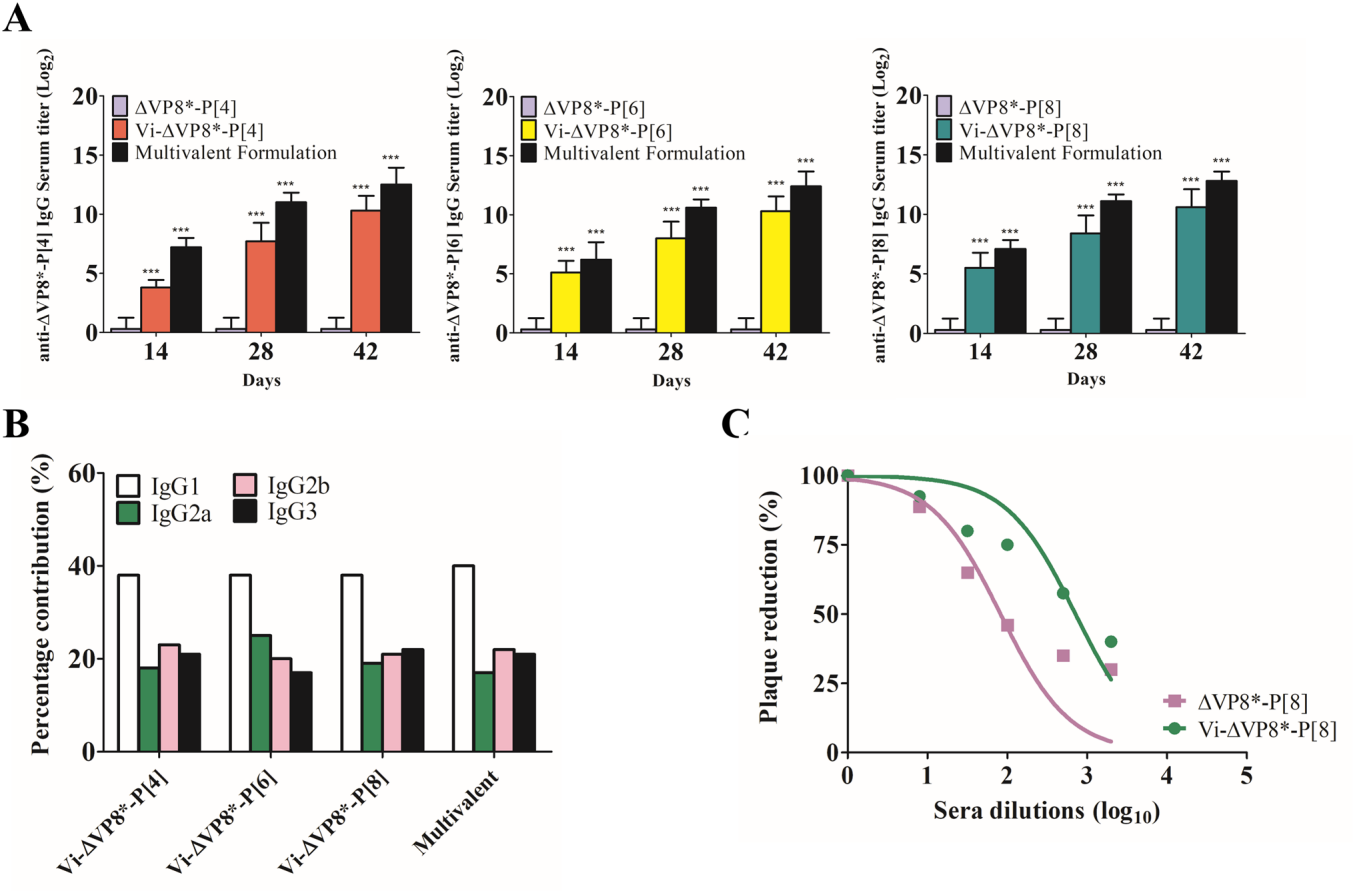
Immunogenicity study of Vi-ΔVP8* for anti-ΔVP8*. (**A**) IgG of mice immunized with ΔVP8* or Vi-ΔVP8* conjugates. (**B**) percentage contribution of IgG subclasses to total IgG against ΔVP8*. (**C**) Neutralization analysis of sera from immunized mice; Day 42 immune sera for titers were used. BALB/c mice were immunized S.C. with 5 μg of Vi-ΔVP8*-P[4], P[6] or P[8], or 15 μg of multivalent formulation based on Vi polysaccharide, 3 times at 2-week intervals as described in Methods. Data were analyzed and illustrated using GraphPad Prism 6.0 software (GraphPad, https://graphpad.com); data are mean±s.d.; two sample *t*-test; *P<0.05, **P<0.01, *** *P*<0.001.

We assessed the levels of IgG antibodies to ΔVP8* elicited by immunization, and neutralizing capacity of serum antibodies was measured using the plaque reduction neutralizing test (PRNT). As previously reported^17,41^, ΔVP8* induced low levels of serum anti-ΔVP8* IgG (Fig. 5A). In contrast, the Vi-ΔVP8* induced high levels of IgG specific for ΔVP8*, and the levels consistently increased through Day 42, with IgG1 being the dominant isotype (Fig. 5A,B). Rotavirus neutralizing activity was higher in conjugate-immunized mice, with 50% reduction in plaque count (PRNT_50_) titers ranging from 1:128 to 1:512 (Fig. 5C).

## Discussion

Vaccination is the most successful strategy to control many infectious diseases. Among several vaccine platforms, conjugate vaccines have dramatically reduced bacterial infections in high-risk groups such as infants and children under two years of age^42,43^. A carrier protein in conjugate vaccines serves as an immune stimulator to a covalently linked polysaccharide antigen, resulting in better immune response in infants and children^44,45^. Among carrier proteins currently used in licensed conjugate vaccines, non-typeable *Haemophilus influenzae* protein D (PD) induces antibody responses not only to a polysaccharide antigen but also to itself. For example, PD in the pneumococcal vaccine Synflorix (GSK) showed a 35.6% protection rate against acute otitis media caused by *Haemophilus influenzae*^46^. While the additional advantage of carrier protein as an immunogen was reported in numerous literatures, sufficient data for its immunogenic potential has not been accumulated^25^.

Our previous studies exploring the conjugate vaccine platform using Vi conjugated to carrier proteins interestingly showed that some carrier proteins play the dual role as a carrier protein and a protective antigen^28,40,47^. In this study, we further demonstrated the potential of Vi conjugation to enhance immune responses in mice to carrier protein compared with those induced by the native protein. For this aim, we produced ΔVP8* antigens from three predominant P rotaviruses and developed Vi-ΔVP8* conjugates by using different linkers and coupling methods, with ADH being the linker of choice. The multiple activation sites of each Vi and multiple linkage points of ΔVP8* allowed us to produce conjugates of large molecular weight. The Vi-ΔVP8* conjugates were carefully characterized, including SEC-HPLC for its molecular size, Hestrin assay for Vi contents, and Bradford assay for protein contents. After analytical assay, three Vi-ΔVP8* conjugates displaying the ΔVP8* antigens of the P[4], P[6], and P[8] specificity were mixed as a multivalent vaccine. Immunization with the Vi-ΔVP8* conjugates in mice significantly increased the immunogenicity of ΔVP8* and induced the T cell-dependent humoral immune response to the Vi. Although multivalent formulated vaccines contained three times higher amount of Vi antigen than in each Vi-ΔVP8* conjugate, the anti-Vi IgG antibody titers were similar. This was similar to the data seen with our Vi-DT conjugate vaccine during its development where the relationship between antigen load and immune response was observed, and up to 10 μg per dose of Vi antigen led to increasing immune response, beyond which it was seen to decrease (Supplementary Fig. S5)^48^. The Vi-ΔVP8* conjugates induced peak anti-Vi IgG responses after one immunization. However, the titers obtained were not enhanced by booster doses, but was maintained even after 3 doses. In a study comparing the short and long Vi polysaccharide conjugated to carrier proteins, the long-chain (165 kDa) Vi conjugates induced higher anti-Vi IgG titers than the conjugates prepared with Vi sizes of less than 82 kDa after the first immunization. However, a booster response after the subsequent immunization was absent with the long-chain Vi conjugates^49,50^. Recent findings by Micoli (*et al.*). demonstrate that polysaccharide length in conjugates plays a major role in eliciting T cell-dependent and T cell-independent immune response^49^. The conjugates with long-chain polysaccharide induced a combined T-cell dependent/independent immune response, whereas the conjugates with short-chain polysaccharides elicited T-cell dependent immune response. In addition, Vi-CRM_197_ induced peak anti-Vi IgG response after the first dose, without a booster response following the second dose in clinical trials^31,51^. Alternatively, maintenance of anti-Vi IgG antibody may result from the defining characteristics of Vi polysaccharide, such as resistance to degradation (*in vivo*) and its large size containing repetitive epitopes (Supplementary Fig. S6). The prolonged persistence of Vi polysaccharide (*in vivo*) may result in continuous induction of polysaccharide-specific, short-lived plasma cells in spleen^52^.

ELISA is widely used to measure the antigen recognition capacities of antibodies in serum, but it may not provide the functional capacities of antibodies. SBA is often used to measure functional antibodies capable of complement-mediated bacterial killing against various bacterial pathogens including cholera, typhoid and meningococcal disease. With meningococcal purified polysaccharide and polysaccharide-protein conjugate vaccines, SBA is accepted as a correlate of protection. Furthermore, the SBA titers showed a strong correlation by linear regression analysis with meningococcal polysaccharide-specific IgG and IgM antibodies^53^. Although there are no standardized assays for assessing functional activities of antibodies to Vi-containing vaccines, a strong and significant correlation was observed between the SBA titers and anti-Vi IgG, which mediates protective immunity to *S*. Typhi^54–57^. Vi polysaccharides are in general T-cell independent type 2 antigens, implying an absence of affinity maturation through somatic hypermutation in germinal centers, immunoglobulin class-switching, and induction of memory B cells; antibodies produced in response to polysaccharide antigens are predominantly IgM^58–60^. IgM may exhibit higher avidity for the antigen than the IgG due to the polyvalent nature of IgM^61^ and are highly effective in killing *S*. Typhi via the classical complement pathway^62,63^. Anti Vi-IgM quantity also showed a moderate correlation with SBA titers (r=0.532, P<0.05)^64^. Therefore, IgM antibodies not quantitated by the IgG ELISA may lead to comparable SBA titers between Vi only and Vi-ΔVP8* conjugates (Fig. 4D).

The multivalent vaccine elicited higher IgG titers to ΔVP8* from P[4], P[6] and P[8] strains as compared to each Vi-ΔVP8* conjugate, though not in a proportionate fashion. Although the saturation limit for ΔVP8* needs to be checked in further studies, this may be explained by the sequence alignments among the three ΔVP8*s; VP8*-P[4] and P[8] share sequence identity of 82%, VP8*-P[6] and P[8] share sequence identity of 63%, and VP8*-P[4] and P[6] share sequence identity of 60%. In addition, the Vi-ΔVP8* conjugates were capable of eliciting functional antibodies against RV and *S*. Typhi. Increased titers of IgG antibodies and serum neutralizing antibodies may lead to protection against both pathogens through serum antibody transudation into the intestinal lumen^65^.

Affinity-based purification of recombinant proteins has become the method of choice for high-throughput protein production due to potential advantages of affinity tags including ease of use, the high level of purity, and the ability to scale up production^66^. Removal of affinity tags from a purified recombinant protein is generally required for vaccine applications because the presence of the affinity tags may interfere with physicochemical properties and immunogenicity of the recombinant protein^67^. However, the complete removal of affinity tags from the target protein is difficult to achieve. To eliminate problems related to them, it is desirable to develop purification methods for tag-free recombinant proteins. We produced the three types of tag free-ΔVP8* by two steps of ion-exchange chromatography to maintain high purity and high levels of consistency between runs. Immunoblotting and SEC-HPLC analysis revealed that the purified ΔVP8* are similar to the results of previous studies^17,68^.

The effect of homologous carrier proteins on the immunogenicity of the conjugate vaccines is ambiguous. In one study, infants who were previously immunized with TT showed higher levels of protection against *Haemophilus influenzae* type b (Hib) upon a single dose of Hib-TT^69^; however, in another study, priming infants with DT and TT did not develop enhanced immune response against Hib even after two booster doses of the Hib conjugates^70^. In addition, several studies have reported that administration of multivalent conjugate vaccines with the same carrier can result in immune interference and impaired immune response to the polysaccharide due to carrier overload or carrier epitope suppression^71,72^. As an example of carrier epitope suppression, tetravalent pneumococcal TT conjugates were found to suppress the anti-polysaccharide immune response of Hib-TT conjugates^73^. Bystander interference is another negative implication of homologous carrier proteins. For example, the reduced immune response of Hib and hepatitis B virus (HBV) were reported in Hexavac (Sanofi Pasteur) consisting of DT, TT, HBV, inactivated poliovirus, Hib polysaccharide conjugated to TT and acellular pertussis^74^. Therefore, the need for a novel carrier protein seems obvious despite the availability of carrier proteins currently used in licensed vaccines. Among several candidates, rEPA has been successfully used as a carrier for *Shigella* O-specific polysaccharide and Vi. The protective efficacy of the conjugates was 74% against *Shigella sonnei* shigellosis and 90% against typhoid fever^75,76^. In addition, novel polyepitope carrier proteins, consisting of a sequential string of universal human CD4^+^ T helper epitopes, exert a strong carrier effect on inducing anti-polysaccharide serum antibody titers and bactericidal activity of antibodies elicited against *Neisseria meningitidis*^77,78^. ΔVP8* plays key roles in rotavirus immune response, inducing effective levels of homotypic and varying titers of heterotypic rotavirus neutralizing antibodies^79,80^. In addition, ΔVP8* significantly improved immunogenicity of the antigen components in the fusion of two or three proteins, thus demonstrating its high potential as a subunit vaccine^16,22^. These data strengthen our conviction to use VP8* as a novel carrier protein in a conjugate vaccine platform.

Parenterally administered Vi-ΔVP8* vaccines may have advantages over currently licensed live oral vaccines due to their potential for concomitant use with other vaccines. Specifically, coformulation of Vi-ΔVP8* vaccines with Expanded Program on Immunization (EPI) vaccines may lower the delivery cost and increase compliance of the vaccination in endemic countries. For example, Vi-CRM_197_ can be given to infants concomitantly with Diphtheria-Tetanus-Pertussis (DTP) vaccine starting at 6 weeks or with measles vaccine at 9 months. The Clinical trial of a Vi-CRM_197_ vaccine in infants demonstrated that the vaccinated individuals showed higher levels of anti-Vi antibodies as compared to the pre-immune level of anti-Vi antibodies^31^. In addition, studies of the Vi-rEPA vaccine showed that the conjugate vaccine was safe and induced a protective immunogenic response when it was used concomitantly with EPI vaccines^81^.

In another perspective, this work opens possibilities for the use of ΔVP8* as a carrier protein in a conjugate platform. Specifically, we are considering conjugation of ΔVP8* to O-specific polysaccharide from *Shigella,* because RV and *Shigella* are invasive enteric pathogens that are strongly associated with acute diarrhea in infants subjected to malnutrition^82,83^. Since there is no licensed vaccine currently available to prevent *Shigella*, the development of a conjugate vaccine could be a significant step towards a safe and efficacious vaccine against both pathogens. Moreover, ΔVP8* can be used in other vaccine platforms; antigen display systems such as VLPs^84^ or those designed by nanotechnology^85,86^ can improve the humoral immune response to ΔVP8*. Among all approaches to develop a subunit vaccine candidate against Rotavirus, recombinant fusion proteins (ΔVP8* fused to a universal tetanus toxin CD4^+^ T cell epitope P2) has been the first to reach clinical trials^87^.

A major limitation of our study is that a challenge study in animal model remains to be elucidated. *In vitro* assays to measure the functional capacities of antibodies are considered as a useful marker associated with protection against mucosal bacterial pathogens. However, animal challenge models provide a more accurate evaluation of vaccine effectiveness and additional information such as the most efficient vaccine formulation, optimal vaccine doses, the optimal route of delivery and immunization schedule. The preclinical data derived from animal challenge models provides an invaluable information to support clinical development strategy.

Additional investigations are required to explore the full potential of multivalent Vi-ΔVP8* vaccine efficacy. Firstly, scale-up from shake flasks to large-scale fermentations allows for high-yield production of ΔVP8* in *E. coli*. In addition, further optimization of conjugation methods can lead to high conjugation efficiency and product consistency with target characteristics. These involve multiple strategies which will result in production of conjugate vaccines at low cost and eventually support its technology transfer to manufacturers in developing countries. Secondly, the mouse sera after immunization with the Vi-ΔVP8*-P[8] showed high neutralizing titers against Wa strain rotavirus in cell culture. Ideally, we should examine the sera from mice immunized with Vi-ΔVP8*-P[4], Vi-ΔVP8*-P[6], or multivalent Vi-ΔVP8* to determine neutralizing activity and cross-neutralizing activity against homologous and heterologous P types of rotaviruses for a comprehensive understanding of the broad efficacy of our multivalent vaccine. This could be part of further evaluation of the vaccine candidates during post-optimization of their manufacturing process. In addition, use of adjuvants in the formulations of conjugate vaccines is also being planned to potentiate the immune response to the vaccine, as adjuvants have shown to induce an enhanced, prolonged and sustainable immune response^88^.

Our work highlights the potential of the conjugate vaccine platform to enhance the immunogenicity of a truncated rotavirus spike protein ΔVP8* that generally induces low immune responses. As a proof of concept, Vi is conjugated to ΔVP8* of P[4], P[6] and P[8] rotaviruses. Mouse immunization studies demonstrated the significantly improved immune responses to ΔVP8* of Vi-ΔVP8* vaccines. Immune responses induced by Vi-ΔVP8* include high titers of Vi-specific serum IgG antibodies and functional antibodies. Our data collectively indicated that highly immunogenic Vi-ΔVP8* conjugates show excellent promise as vaccine candidates which can tackle two significant enteric pathogens, and that a conjugate vaccine platform using Vi has the potential to enhance the immunogenicity of carrier proteins.

## Methods

### Plasmid constructs

DNA sequences encoded for amino acids 64 to 223 of VP8*-P[4], 64 to 223 of VP8*-P[6], or 65 to 223 of VP8*-P[8] were synthesized by Bioneer (Daejeon, Korea) and cloned into the expression vector pET28a (Novagen, USA) for protein expression. Amplification of the recombinant plasmids was conducted by transformation into *Escherichia coli* (*E. coli*) (strain BL21, DE3).

### Recombinant protein production

Recombinant proteins were expressed in *E. coli* BL21 (DE3) as described previously^17^. Briefly, *E. coli* BL21 (DE3) cells harboring the expression vectors were grown at 37°C until absorbance at 600 nm reached 0.6. The expression of tag-free ΔVP8* proteins were induced by the addition of 0.5 mM isopropyl-β-D-thiogalactopyranoside (IPTG) at 18°C overnight. The recombinant *E. coli* cells harboring ΔVP8* proteins were collected by centrifugation and lysed by sonication into 20 mM Tris buffer (pH7.6). Purification of the proteins was carried out using resins of ion exchangers (Merck) according to manufacturer’s instructions.

### SDS-PAGE and immunoblot

Quality of the purified proteins were analyzed by sodium dodecyl sulfate polyacrylamide gel electrophoresis (SDS-PAGE) using 12% separating gels, followed by immunoblotting. The gels were stained with SimplyBlue Safestain Solution (Invitrogen, USA). For immunoblotting, the proteins were transferred to a 0.2 μm nitrocellulose membrane using Trans-Blot Turbo Transfer pack and Trans-Blot Turbo Blotting System (Bio-Rad, USA). The membrane was block with Tris-buffered saline (TBS) containing 5% non-fat milk (w/v) and incubated at room temperature for 1 h followed by rinsing three times with TBS with 0.1% Tween 20 (TBS-T). The membrane was applied at 4 °C overnight with anti-rotavirus polyclonal antibody (AB1129; Merck, Germany) and followed by rabbit anti-goat IgG conjugate (Southern Biotech, USA). Immunoreactive proteins were detected using enhanced chemiluminescence assay (ECL) (Thermo Scientific, USA). The original gel and blot images are provided in ‘Supplementary Figures’.

### Purification of recombinant proteins

Ion exchange chromatography using anion exchange resin (AEX), Fractogel EMD DEAE (Merck, Germany) resin in XK16/20 column with 16 cm bed height, was performed in negative (flow-through) mode to separate the ΔVP8* proteins from impurities such as host cell proteins and DNA. The unbound proteins were concentrated and then diafiltered into 20 mM Sodium Acetate (pH5) buffer by Tangential Flow Filtration (TFF) using 5 kDa molecular weight cut-off (MWCO) membrane cassettes for further purification. Subsequently, 5 kDa retentate was loaded on the cation exchange chromatography (CEX) using Eshmuno CPX (Merck, Germany) resin in XK16/20 column with 15 cm bed height, followed by washing of the column using 5 CVs of 20 mM Sodium Acetate (pH5) buffer to remove unbound proteins. The bound proteins were eluted using 10 CVs of 20 mM Sodium Acetate buffer with 1 M NaCl (pH5). For further application, the eluted fractions containing ΔVP8* proteins were concentrated and diafiltered into phosphate buffered saline (PBS, pH7.4) by TFF using 5 kDa membrane cassettes. The protein concentration of the samples was determined by Bradford assay. The average size of the purified ΔVP8* proteins was determined by High performance size exclusion liquid chromatograph (SEC-HPLC; Agilent 1260 LC system, USA) equipped with a OHpak SB-804 HQ column (Shodex, Japan). Quantity of the purified protein was measured by Bradford method using bovine serum albumin (BSA) as a standard. Endotoxin levels and DNA quantification were performed using Endosafe nexgen-PTS spectrophotometer (Charles River, USA) and Quant-iT Picogreen dsDNA assay kit (Invitrogen, USA), respectively.

### Preparation of Vi-ΔVP8* conjugates

Briefly, aspartic or glutamic acid residues of the purified ΔVP8* were modified with adipic acid dihydrazide (ADH) in the presence of 1-ethyl-3-(3-dimethylaminopropyl)carbodiimide (EDAC) to achieve an efficient coupling of ΔVP8*^89^. The reaction was performed in MES buffer (80 mM, pH5.1) at room temperature for 1 h, and then the excess linkers were removed by spin desalting column. The derivatized VP8* (ΔVP8*_AH_) was filter sterilized through 0.2 μm syringe filter. Protein content and free hydrazide content were determined by Bradford assay and 2,4,6-trinitrobenzene sulfonic acid (TNBS) assay, respectively.

Vi-ΔVP8* conjugates were prepared as previously described^28,40^. 8 mg of Vi was dissolved in 4 mL of MES buffer (80 mM, pH 4.9), and 250 μL of 50 mg/mL EDAC was added and mixed with agitation for 5 min. at room temperature. Then 8 mg of ΔVP8* was added to the mixture, and the reaction was performed with agitation at room temperature for 2 h. The mixture was then dialyzed in PBS for 2 days. To isolate the conjugates from the free Vi or ΔVP8*_AH_, the dialyzed mixture was separated using Fast protein liquid chromatography (FPLC) on a Sephacryl S-1000 (GE Healthcare, USA) resin in XK16/100 column with 90 cm bed height. Each fraction was then assayed for polysaccharide and protein contents by Hesterin and Bradford assays, respectively. The average size of the Vi-ΔVP8* conjugates was determined using SEC-HPLC (Agilent 1260 LC system, USA) equipped with OHpak SB-804 HQ and OHpak SB-806 HQ columns in series (Shodex, Japan).

### Mouse immunization

BALB/c mice at about six weeks of age were purchased from KOATECH (Korea) and maintained under specific pathogen-free conditions at the Animal facility of International Vaccine Institute (IVI). They were randomly divided into 9 groups of 10 mice each (*N*= 10), and each group was immunized with one of the following immunogens: (1) a mixture of the three Vi-ΔVP8* conjugates, representing P[4], P[6] and P[8] rotaviruses, in equal amount of Vi (5 μg each) as a multivalent vaccine at 3.6 μg total ΔVP8*/mouse/dose; (2) the Vi-ΔVP8* of P[4] type at 1.0 μg ΔVP8*/mouse/dose; (3) the Vi-ΔVP8* of P[6] type at 1.1 μg ΔVP8*/mouse/dose; (4) the Vi-ΔVP8* of P[8] type at 1.5 μg ΔVP8*/mouse/dose; (5) ΔVP8* of P[4] type at 1.0 μg/mouse/dose; (6) ΔVP8* of P[6] type at 1.1 μg/mouse/dose; (7) ΔVP8* of P[8] type at 1.5 μg/mouse/dose; (8) the Vi polysaccharide without the ΔVP8* antigens at 5 μg/mouse/dose as a control; and (9) PBS as vaccine diluent control. Immunogens in 150μL volumes were administered subcutaneously three times at 2-week intervals. Blood samples were taken at 14, 28, and 42 days from retro-orbital bleeding, allowing to clot for 30 min., and centrifuged at 1,200xg at 4°C. All animal studies were carried out as per the guidelines and following approval from Institutional Animal Care and Use Committee (IACUC) at International Vaccine Institute (IVI; Seoul, Korea). Experiments were designed and reported with reference to the Animal Research: Reporting of *in vivo* Experiments (ARRIVE) guidelines^90^.

### Enzyme linked immunosorbent assays (ELISA)

ELISA was utilized to define Vi- or ΔVP8*-specific antibody titers using the method previously described^48^. Briefly, Vi polysaccharide or ΔVP8* proteins of P[4], P[6] and P[8] rotaviruses were coated on 96-well plates at 1 μg/mL. After blocking with 1% (w/v) BSA, plates were incubated with mouse sera at serial twofold dilutions. Bound antibodies were measured by goat-anti-mouse IgG-Alkaline phosphate conjugate (1:5,000; Southern Biotechnologies, USA). Antibody titers were described as the maximum dilution of sera that exhibited at least cut-off signals of OD_450_ = 0.15.

### Plaque reduction neutralization assays

A virus neutralization assay based on plaque reduction was performed to determine neutralizing antibodies elicited in vaccinated mice. Briefly, MA104 cells were cultivated in six-well plates. After a treatment with trypsin (10 μg/mL in Medium 199), rotaviruses of P[8] (Wa strain, G1P8) were incubated with mouse sera at given dilutions, and then added to MA104 cell monolayers in 6-well plates and incubated for 1 h at 37°C on a rocking platform. The cells were washed with prewarmed, serum-free Medium 199, gently overlaid with 0.7% agarose and incubated at 37°C. The cells were fixed with 10% formalin and stained with 0.5% crystal violet. The neutralization titers of the sera were defined as the maximum dilutions of the mouse sera that can reduce at least 50% of infected cells compared with non-neutralized, serum free control as previously described^91^.

### Statistical analysis

Statistical differences among data sets were determined and presented by GraphPad Prism 6 (GraphPad Software, Inc, USA; https://graphpad.com) through a paired *t* test. Differences were considered non-significant (ns) for p-values >0.05 (marked as “ns”), significant for p-values <0.05 (marked as *), highly significant for p-values <0.01 (marked as **) and extremely significant for p-values <0.001 (marked as ***), respectively.

## Supplementary Information

Supplementary Information.
