# Supplementary material for: Rotavirus spike protein ΔVP8* as a novel carrier protein for conjugate vaccine platform with demonstrated antigenic potential for use as bivalent vaccine
Source: Sci Rep. 2021 Nov 11;11:22037. doi: 10.1038/s41598-021-01549-z (PMC8586335; doi:10.1038/s41598-021-01549-z)
Supplement: Supplementary file 1 — Supplementary Information. [file 41598_2021_1549_MOESM1_ESM.pdf]

Rotavirus spike protein ΔVP8* as a novel carrier protein for conjugate vaccine platform with demonstrated antigenic potential for use as bivalent vaccine

Wook-Jin Park[1,2], Yeon-Kyung Yoon[2], Ji-Sun Park[2], Ruchirkumar Pansuriya[2], Yeong-Jae Seok[1] and Ravi Ganapathy[2,*]

[1]School of Biological Sciences and Institute of Microbiology, Seoul National University, Seoul 08826, Republic of Korea

[2]International Vaccine Institute, Seoul 08826, Republic of Korea

*Corresponding: ravi.ganapathy@ivi.int

Supplementary Figures

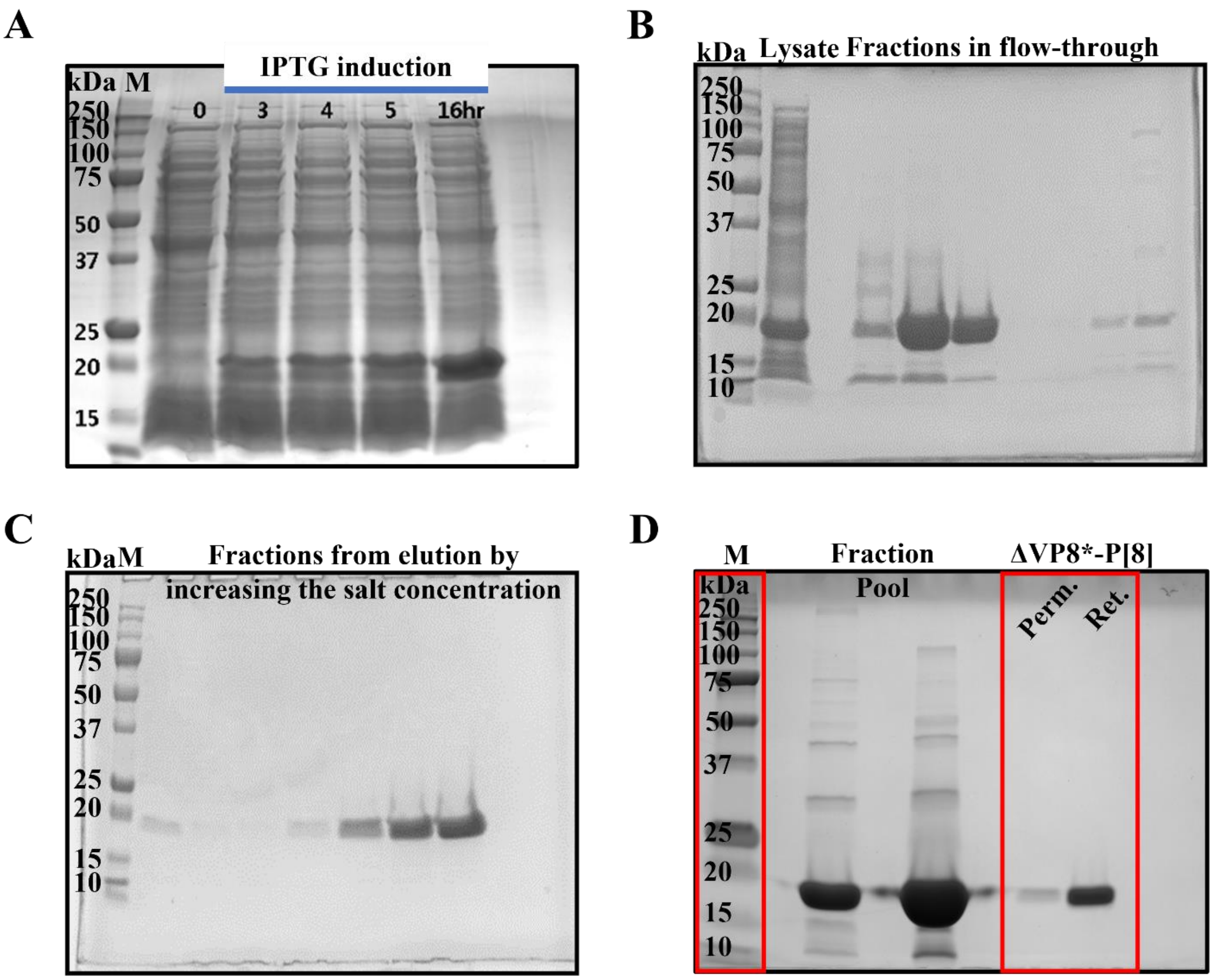

Supplementary Figure 1. Expression and purification the recombinant ΔVP8*-P[8] protein. (A) SDS-PAGE profiles of the expression of ΔVP8* upon IPTG induction. SDS-PAGE analysis result of (B) the anion exchange chromatography, (C) the cation exchange chromatography, and (D) concentration and diafiltration using 5kDa cut-off membrane (Ret.: Retentate from 5 kDa TFF, Perm.: Permeate from 5 kDa TFF, and M: Marker). Red boxes indicate the region selected for presentation in Figure 1A. Full-size gels were imaged with the imaging Densitometer (Model GS-800, Bio-Rad). SimplyBlue stained 12% SDS-PAGE.

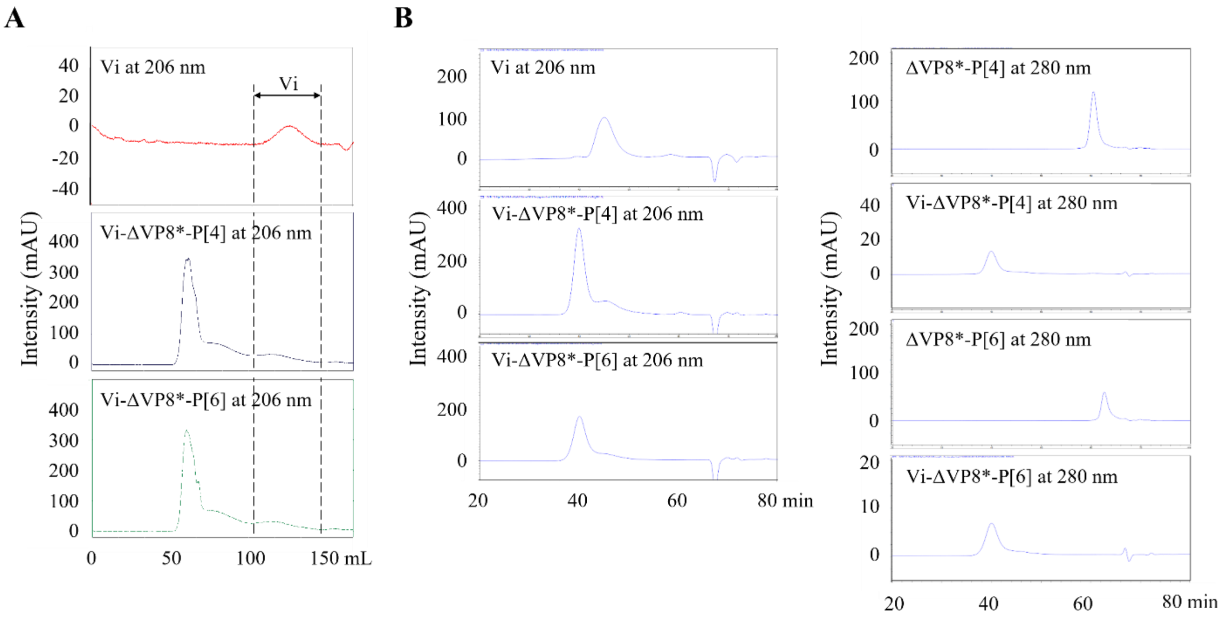

Supplementary Figure 2. Physicochemical characteristics of the glycoconjugates. (A) Sephacryl S-1000 profiles of Vi and Vi-ΔVP8*-P[4] and P[6]. Vi and Vi-ΔVP8* conjugates was dialyzed against PBS and loaded onto Sephacryl S-1000 (1.6cm × 90cm) eluting with 10mM NaH_2PO_4, 5mM NaCl, pH 7.0 at a flow rate of 0.5 mL/min. (B) SEC-HPLC profiles of Vi-ΔVP8*-P[4] and P[6]. OHpak SB-804 HQ and OHpak SB-806 HQ columns in series, 0.1M NaCl, 0.1M NaH_2PO_4, 5% ACN, pH 7.2; 0.3 mL/min. flow rate.

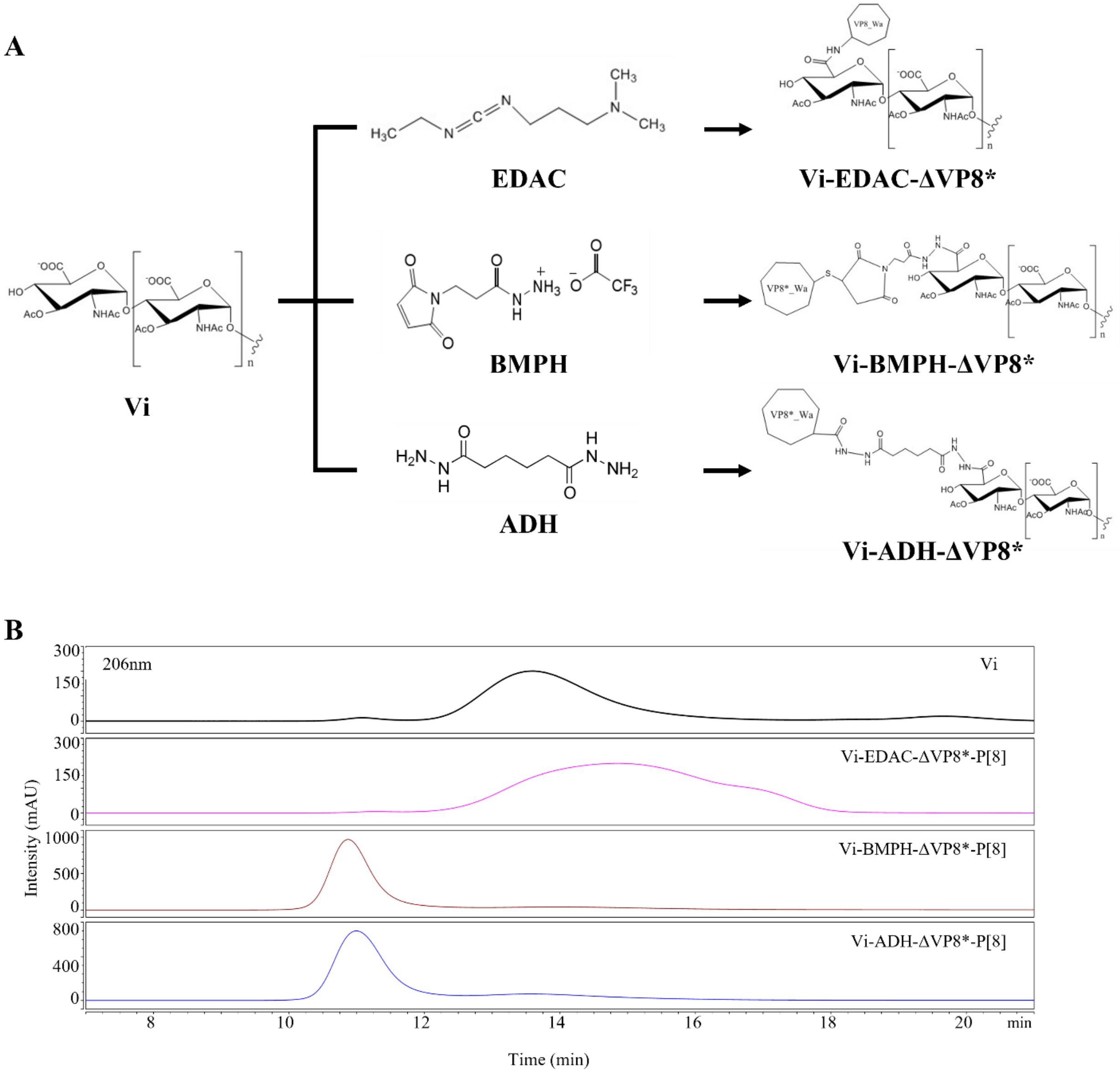

Supplementary Figure 3. Conjugation scheme and physicochemical characteristics of Vi-ΔVP8* conjugates with different linkers. (A) Conjugation scheme for the synthesis of Vi-ΔVP8* conjugates. (B) SEC-HPLC analysis of Vi-ΔVP8*-P[8] conjugates

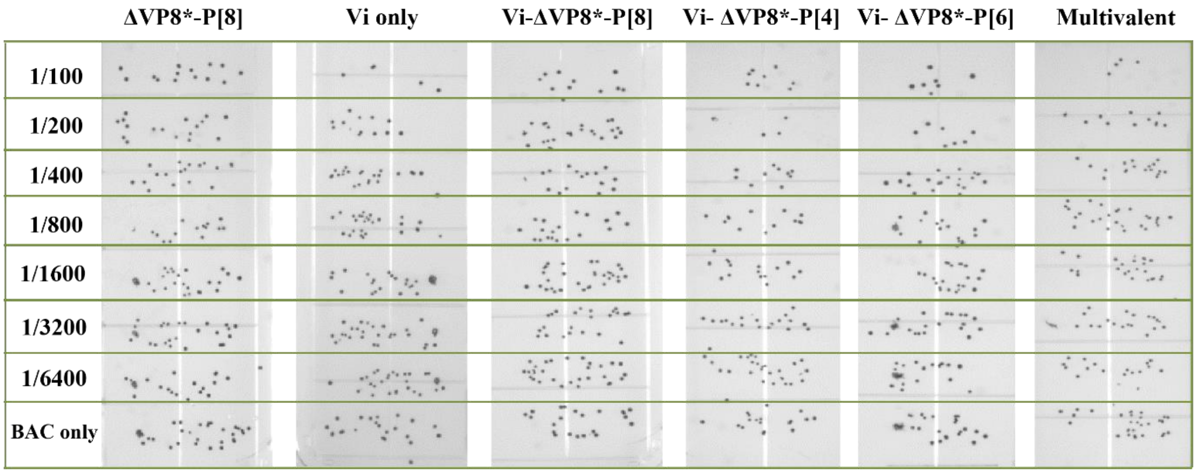

Supplementary Figure 4. SBA measures functional *S*. Typhi specific antibodies capable of complement-mediated bacterial killing.

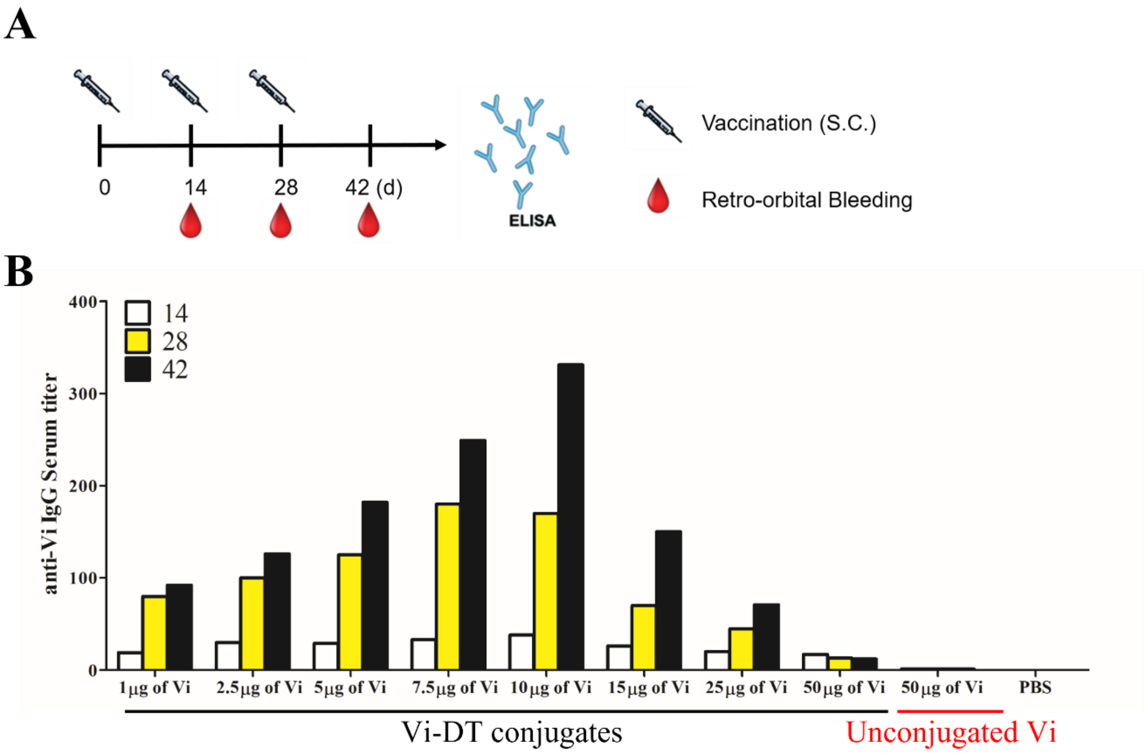

Supplementary Figure 5. Influence of Vi amount on the immunogencity of Vi conjugates in mice. (A) A schematic diagram for subcutaneous (SC) vaccination and bleeding. (B) Anti-Vi IgG antibody titer. Mice were immunized with three doses of Vi-DT conjugates at 1 μg, 2.5 μg, 5 μg, 7.5 μg, 10 μg, 15 μg, 25 μg, or 50 μg Vi/dose. Mice were injected with 50 μg of Vi. Controls were injected with PBS.

A

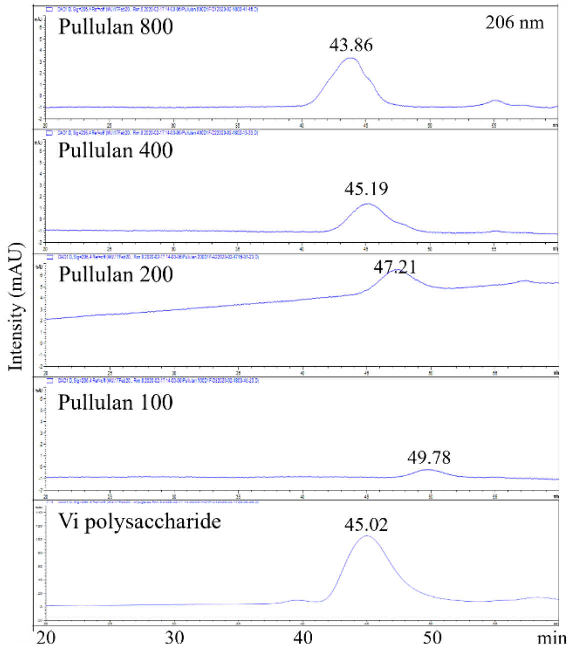

B

Standard	Retention time (Min)	Mw (kDa)
Pullulan 800	43.86	736
Pullulan 400	45.19	343
Pullulan 200	47.21	202
Pullulan 100	49.78	110
**Sample**	**Retention time (Min)**	**Mw (kDa)**
Vi polysaccharide	45.02	440

Supplementary Figure 6. SEC-HPLC profiles of pullulan standard and Vi. OHpak SB-804 HQ and OHpak SB-806 HQ columns in series, 0.1M NaCl, 0.1M NaH_2PO_4, 5% ACN, pH 7.2; 0.3 mL/min. flow rate. B. The molecular weight of Vi polysaccharide was calculated based on pullulan standards.

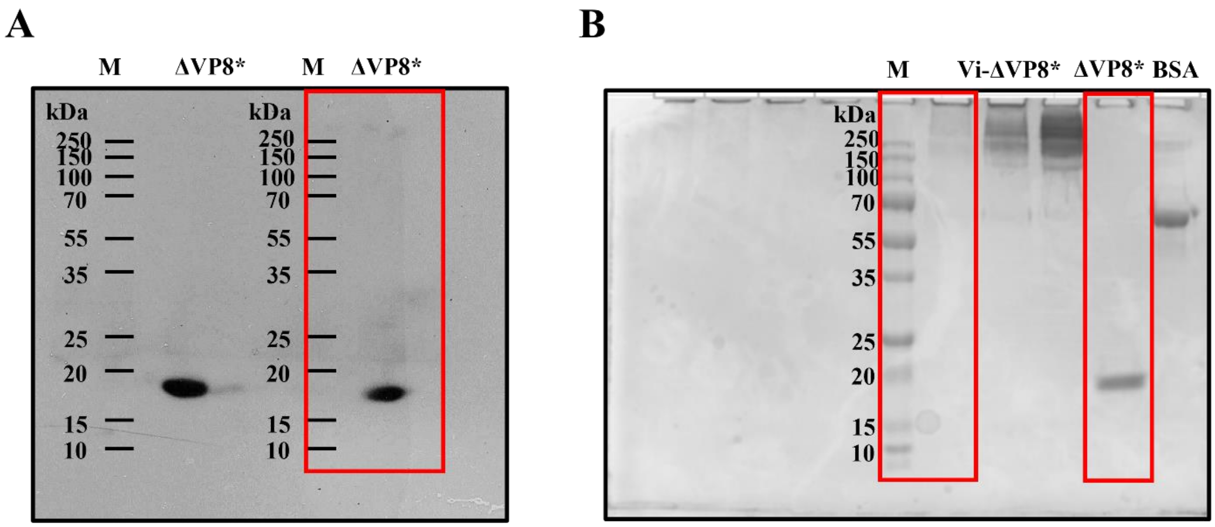

Supplementary Figure 7. Original blot and gel referring to main Figure 1A and Figure 3C, respectively. (A) Red box indicates the region selected for presentation in Figure 1A. (B) Red boxes indicate the region selected for presentation in Figure 3C. Full-size Western Blot membrane and gel were imaged with the imaging Densitometer (Model GS-800, Bio-Rad)

Supplementary Table 1. Chemical analysis of conjugates prepared by different linkers.

Conjugate	Linker	Recovery (%)[1]	
		Polysaccharide[2]	Protein[3]
Vi-EDAC-ΔVP8*-P[8]	EDAC	0	0
Vi-BMPH-ΔVP8*-P[8]	BMPH	40	5
Vi-ADH-ΔVP8*-P[8]	ADH	44	15

[1]Recovery of each conjugate obtained was calculated on the basis of Vi concentration and protein concentration.
[2]Vi concentration was based on Hestrin assay.
[3]ΔVP8* protein concentration was based on Bradford assay.
